# Predictors of initial oral food challenge outcome in food protein–induced enterocolitis syndrome

**DOI:** 10.1016/j.jacig.2022.05.004

**Published:** 2022-07-11

**Authors:** Satoshi Hayano, Osamu Natsume, Ryuhei Yasuoka, Yukiko Katoh, Masaki Koda

**Affiliations:** a Allergic Disease Research Center, Chutoen General Medical Center, Kakegawa, Japan; b Department of Pediatrics, Chutoen General Medical Center, Kakegawa, Japan; c Department of Pediatrics, Hamamatsu University School of Medicine, Hamamatsu City, Japan; d Department of Pediatrics, Public Morimachi Hospital, Morimachi, Japan

**Keywords:** Diagnosis, food allergy, food protein–induced enterocolitis syndrome, oral food challenge

## Abstract

**Background:**

There is a paucity of data on predictors of clinical history in oral food challenge (OFC) outcome for the initial diagnosis of food protein–induced enterocolitis syndrome (FPIES).

**Objective:**

This study aimed to identify predictors for the diagnosis of FPIES.

**Methods:**

The study included patients who underwent OFC to diagnose FPIES from 2010 to 2021. Patients with a positive OFC result were classified as belonging to the FPIES group, and those with negative OFC result within 120 days from the last symptomatic episode were classified as belonging to the no-allergy (NA) group. Background factors were analyzed in the groups.

**Results:**

A total of 50 OFCs to 12 different foods were conducted in 50 patients. Of those 50 patients, 30 were classified as belonging to the FPIES group. No significant difference was observed between the FPIES and NA groups with respect to background factors, including the features of symptomatic episodes and examinations of immediate-type allergy. A history of asymptomatic ingestion was observed in 23 of 24 and 13 of 19 patients in the FPIES and NA groups, respectively; thus, it was significantly more common in patients with FPIES. The diagnostic rate of patients with fewer than 3 symptomatic episodes was 52%, and that of patients with 3 episodes or more was 75%, not considering a patient without available data.

**Conclusions:**

A definite diagnosis of FPIES should be based on OFC, as there are no predictors for OFC positivity other than a history of asymptomatic ingestion. The absence of asymptomatic ingestion history was a negative predictor for the diagnosis of FPIES.

Food protein–induced enterocolitis syndrome (FPIES) is a non–IgE-dependent food allergy characterized by delayed gastrointestinal symptoms. FPIES has an incidence ranging from 0.34% to 0.51% among children.^1^^,^^2^ FPIES typically causes vomiting 1 to 4 hours after ingestion of the culprit food, occasionally followed by diarrhea or bloody stool. The diagnostic criteria for FPIES was proposed in the 1970s.^3^ The international guidelines proposed by Nowak-Węgrzyn et al in 2017 are currently in use.^4^ The guidelines’ diagnostic criteria for FPIES allow a definitive diagnosis based on clinical history alone, including protracted delayed vomiting as a core symptom.^4^

There is no diagnostic marker for FPIES. In many patients, only nonspecific findings, including neutrophilia, eosinophilia, and thrombocytosis, can be obtained by laboratory tests.^5^ Data on the accuracy of the initial diagnosis of FPIES based on clinical history are also lacking. Most large-scale population studies of FPIES have been based on data without an oral food challenge (OFC)-validated diagnosis.^1^^,^^6^, ^7^, ^8^, ^9^ Although several studies have investigated the positivity rate of OFC for FPIES (which ranged from 10% to 35%),^8^^,^^10^^,^^11^ these studies included OFCs for assessing the development of tolerance but not OFCs for the diagnosis. A recent study also reported the results of as many as 11 of 21 (52%) diagnostic OFCs for FPIES as negative.^12^ Thus, there might be a high false positivity rate when the FPIES diagnosis is based solely on a history of multiple episodes of protracted delayed vomiting, which may impair the patient’s quality of life.

The following 2 clinical questions need to be answered: “Is it appropriate to diagnose FPIES solely on the basis of clinical history?” and “Are there any predictors of FPIES OFC outcome?” Therefore, we conducted a multicenter retrospective case-control study of patients who were suspected of having FPIES, visited a tertiary medical center, and underwent an OFC.

## Methods

### Patient identification

This multicenter retrospective case-control study included data from the patients’ medical records. OFCs were performed on patients attending the tertiary medical centers (Hamamatsu University Hospital, Chutoen General Medical Center, and Public Morimachi Hospital) for suspected FPIES from September 2010 to May 2021. The inclusion criteria were as follows: (1) pediatric patients younger than 15 years, (2) those who met the international consensus guidelines criteria for acute FPIES,^4^ and (3) those who underwent OFC for a diagnosis. The exclusion criteria were (1) patients without protracted delayed vomiting and (2) patients with a negative OFC result after 120 or more days from the last symptomatic episode, because they could not be distinguished from those with spontaneous remission of FPIES and no allergy (NA). The institutional review board of Chutoen General Medical Center approved this study (approval no. MR148). All patients who underwent OFC were given the opportunity to opt out of the study, and those who declined to participate were excluded.

### Procedure

Children who were suspected of having FPIES and visited tertiary hospitals underwent specific IgE testing for the culprit food at the initial visit. A prick-to-prick skin prick test (SPT) for the culprit food was performed before OFC. OFC was recommended for a definitive diagnosis of FPIES. If a patient refused OFC, he or she was instructed to avoid culprit foods of FPIES, and OFC was performed after several months to confirm remission. Patients who had a positive OFC result at any time were included in this analysis as confirmed patients with FPIES. Children with a negative OFC result within 120 days were included in the NA group, whereas those with a negative OFC result after 120 or more days from their last symptomatic episode were excluded because whether they were in spontaneous remission of FPIES or had NA could not be determined. Background factors before the initial OFC were statistically analyzed for patients in the FPIES and NA groups.

### OFC protocol

All patients underwent open OFC. Culprit foods included cow’s milk, cow’s milk formula, hen’s egg yolk, hen’s egg white, soy, wheat, quail’s egg yolk, fish, rice, buckwheat, kiwifruit, and banana. Solid foods were processed to the usual edible form before OFC. Antiallergic medications, including antihistamine agents, antileukotriene agents, and systemic steroids, were stopped before OFC (at 72 hours, 24 hours, and 7 days before OFC, respectively). We modified the OFC protocol of the international guidelines, which call for administering food in 3 equal doses over 30 minutes, followed by the daily intake dose.^4^ An initial OFC was typically performed by using a 3-dose escalation protocol. The starting dose was the same as the dose during the last symptomatic episode, followed by an intermediate dose and an age-appropriate serving dose with a minimum interval of 4 hours. An age-appropriate serving dose was administered as a maximum dose at the provider’s discretion (eg, 15 g of hen’s egg yolk or 100 to 200 mL of cow’s milk). Patients with protracted delayed vomiting within 1 to 4 hours with or without diarrhea within 5 to 10 hours were classified as positive for FPIES in the OFC. Patients with a negative OFC result were instructed to continue taking the maximum safe daily dose at home for a week. If patients with a daily home intake of the culprit food developed protracted delayed vomiting on different days, they were classified as belonging to the FPIES group. OFC-positive patients were classified as belonging to the FPIES group, and those with a negative OFC result were classified as belonging to the NA group.

### Risk factors

We examined whether there was a difference between the FPIES and NA groups based on OFC result. The following data were investigated: age at onset; history of asymptomatic intake before onset; number of symptomatic episodes; interval from ingestion to elicitation; number of vomiting episodes; presence or absence of diarrhea; presence or absence of bloody stool; history of IgE-mediated allergy to other foods and atopic dermatitis (AD) at the first visit; family history of food allergy, AD, and bronchial asthma; total IgE level and positive (≥ 0.35 kUA/L) rate of food-specific IgE test result for the culprit food at first hospital visit (ImmunoCAP, Thermo Fisher Scientific, Uppsala, Sweden); and positive result of an SPT with culprit foods on the day of OFC.

### Definition of factors

Date of onset was defined as the day of the first symptom. Number of vomiting episodes was defined as the maximum number of vomiting episodes during each symptomatic episode reported by the patients. Presence of diarrhea and bloody stool was defined as positive when accompanied by protracted delayed vomiting during symptomatic episodes before OFC. History of IgE-mediated other food allergy was defined as positive based on the clinical history and a positive food-specific IgE test result.

### Statistical analysis

Continuous data are described as medians and interquartile ranges. Predictors associated with a positive OFC result were evaluated by using the Fisher exact test (for a history of asymptomatic ingestion, diarrhea, bloody stool, IgE-mediated food allergy, family history of AD and bronchial asthma, and results of SPTs) and the Wilcoxon rank sum test (for age at the first symptomatic episode, number of symptomatic episodes, interval from ingestion to symptom onset, number of vomiting episodes, and total IgE level). A *P* value less than .05 was considered statistically significant. Not all survey data were complete; thus, the denominators for each data were different. Listwise deletion was used for handling missing data. Sensitivity analysis was performed by treating all OFC-negative cases exceeding 120 days as cases of spontaneous remission of FPIES as an extreme hypothesis. R for Windows version 4.0.5 (R Foundation for Statistical Computing., Vienna, Austria) was used for the statistical analysis.^13^

## Results

### Overview of data

**Fig 1:**
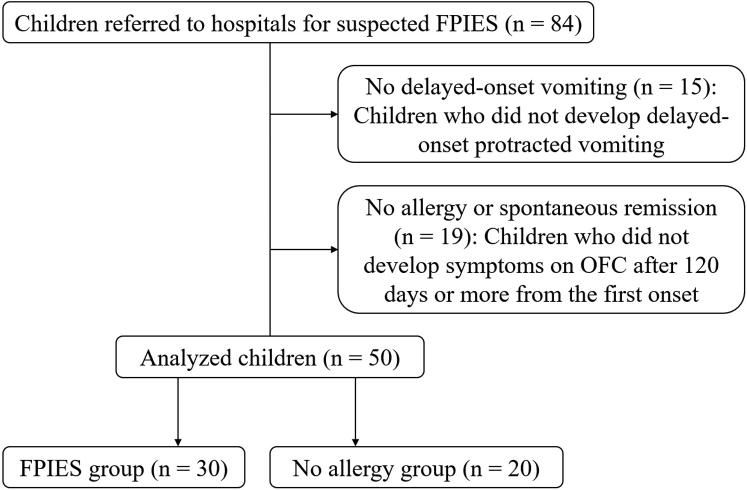
Flowchart of the study population. In all, 83 patients with suspected FPIES were referred to hospitals. Of those patients, 15 and 19 who did not develop protracted delayed vomiting or failed to develop symptoms on OFC after 120 or more days from the first onset, respectively, were excluded. In all, 30 patients were included in the FPIES group, and 20 were included in the NA group.

During the study period, 84 patients suspected of having FPIES were identified (Fig 1). A total of 15 patients were excluded because they did not have protracted delayed vomiting. In all, 19 patients were excluded because they had a negative OFC result after 120 or more days from the last symptomatic episode. Therefore, 50 patients were enrolled: 30 patients were included in the FPIES group as OFC-positive (n = 27) or with symptoms induced at home after OFC (n = 3) and 20 patients were included in the NA group as OFC-negative.

### Challenge foods

**Fig 2:**
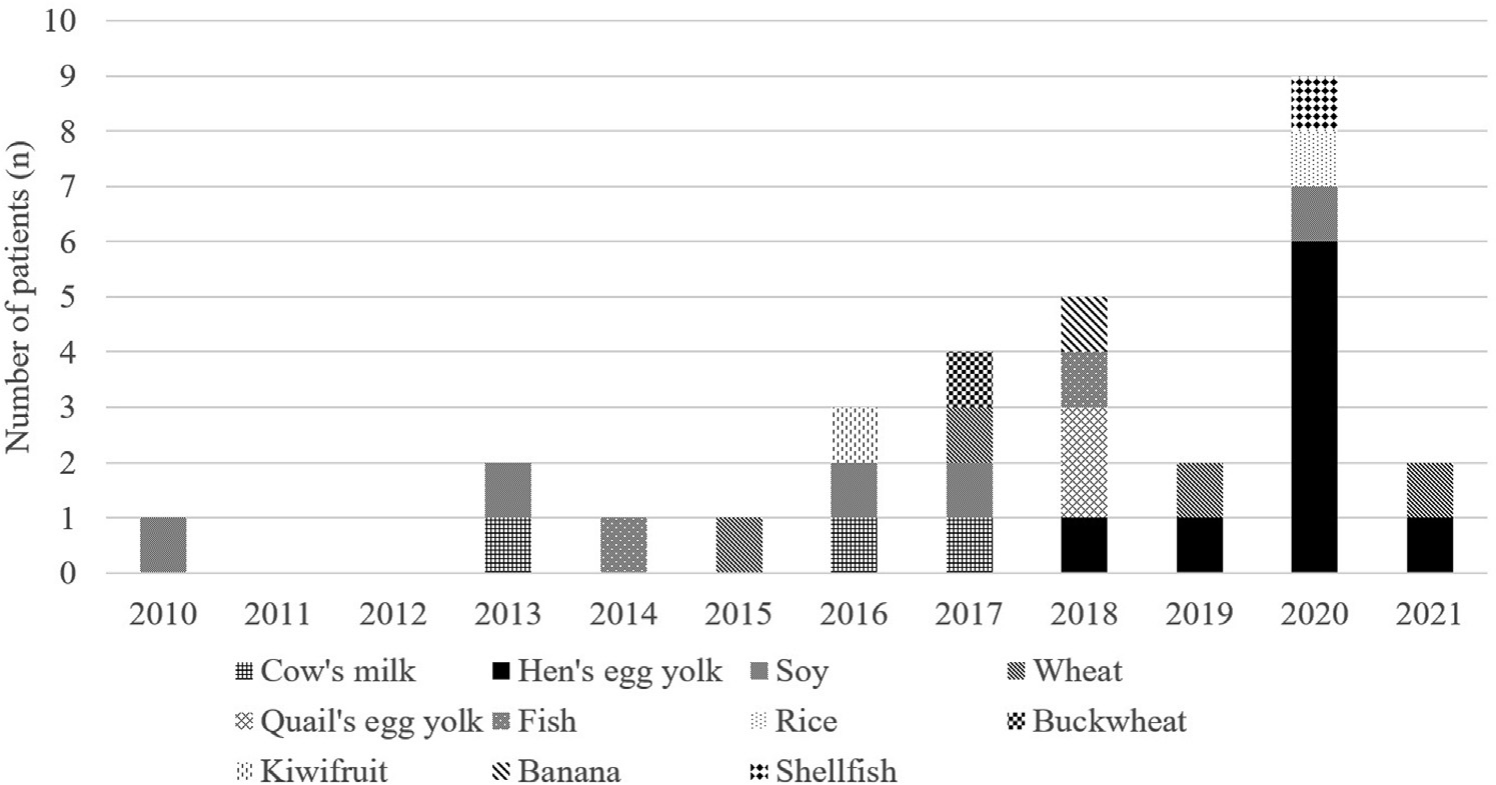
Transition of causative foods of FPIES.

In the FPIES group, 11 culprit foods were found in 30 patients, with hen’s egg yolk, soybean, wheat, and cow’s milk mostly implicated (Table I). In 2 patients in the FPIES group, another food was also suspected of causing the FPIES. One patient with soy FPIES was also suspected of cow’s milk FPIES; the patient had only diarrhea at the initial OFC with cow’s milk and tolerated home dosing after the OFC. Another patient with hen’s egg yolk FPIES was also suspected of having soy FPIES; the patient had no symptoms elicited on the initial OFC with soy 156 days after the last symptomatic episode. Thus, there were no patients with FPIES with multiple trigger foods. In the NA group, 6 types of suspected foods were listed for 20 patients. The culprit food of FPIES shifted from cow’s milk to hen’s egg yolk over time, and there was a wide variety of solid foods other than hen’s egg yolk that could induce FPIES (Fig 2).

**Table I:** Diagnostic rate of foods suspected as causes for FPIES

	FPIES group (n = 30)	NA group (n = 20)	Diagnostic rate (%)
Hen’s egg yolk	9 (31%)	13 (65%)	41
Soy	5 (17%)	2 (10%)	71
Wheat	4 (14%)	1 (5%)	80
Cow’s milk	3 (10%)	2 (10%)	60
Quail’s egg yolk	2 (7%)	0 (0%)	100
Fish	2 (7%)	0 (0%)	100
Rice	1 (3%)	1 (5%)	50
Buckwheat	1 (3%)	0 (0%)	100
Kiwifruit	1 (3%)	0 (0%)	100
Banana	1 (3%)	0 (0%)	100
Shellfish	1 (3%)	0 (0%)	100
Hen’s egg white	0 (0%)	1 (5%)	0

### Risk factors for a positive OFC result

No significant difference was observed between the FPIES and NA groups in any of the data investigated except for asymptomatic ingestion history (*P* = .03) (Table II). There was no difference in age of onset or interval between the last symptomatic episode and OFC (Table II and see Figs E1 and E2 in the Online Repository at www.jaci-global.org). In the FPIES group, 7 of 30 patients underwent OFC more than 120 days after the last episode. The average intervals between the last episode and OFC were 119 and 57 days in the FPIES and NA groups, respectively. Almost all of the patients in the FPIES group with available data had a history of asymptomatic ingestion of culprit foods. The only patient who had FPIES with buckwheat allergy and did not have an asymptomatic ingestion history tolerated small amounts of contamination in a boiled soup of buckwheat before the first symptomatic episode. The absence of a history of asymptomatic ingestion reduced the likelihood of a positive FPIES OFC result, which had a negative predictive value of 86% (6 of 7). The median number of symptomatic episodes was greater in the FPIES group than in the NA group, with no significant difference (*P* = .09) between the 2 groups. The diagnostic rates based on the number of symptomatic episodes were 40%, 54%, 75%, and 75% for 1, 2, 3, and 4 episodes or more, respectively (Table III). There was no significant difference in the specific IgE titers for crude and component antigens, which were positive for some culprit foods (Table IV). Of the patients in the FPIES group who were tested, 3 of 20 (15%) were positive for specific IgE.

**Table II:** Clinical factors associated with FPIES

Factor	FPIES group (n = 30)		NA group (n = 20)		*P* value
	Missing data (n)		Missing data (n)		
Age at the first episode (d), median (IQR)	262 (205-301)	0	221 (211-243)	0	.11
Interval between the last symptomatic episode and OFC (d), median (IQR)	59 (41-117)	1	51 (42-75)	0	.48
History of asymptomatic ingestion (no.)	23 (96%)	6	13 (68%)	1	.03∗
Symptomatic episodes (no.), median (IQR)	2.5 (2.0-3.0)	0	2.0 (2.0-2.5)	1	.09
Ingestion to onset interval (h), median (IQR)	2 (2-3)	4	2 (1-2)	0	.10
Vomiting (no.), median (IQR)	3 (1-5)	1	3.0 (1.8-4.3)	0	.73
Accompanied by diarrhea (no.)	6 of 29 (21%)	1	4 of 20 (20%)	0	1.00
Accompanied by bloody stool (no.)	1 of 29 (3%)	1	0 of 17 (0%)	3	1.00
IgE-mediated allergy to other than the culprit food (no.)	5 of 29 (17%)	1	1 of 20 (5%)	0	.38
Atopic dermatitis (no.)	12 of 29 (41%)	1	8 of 20 (40%)	0	1.00
Family history					
Atopic dermatitis (no.)	4 of 28 (17%)	2	8 of 20 (67%)	0	.09
Bronchial asthma (no.)	9 of 28 (32%)	2	5 of 20 (25%)	0	.75
Total IgE level (IU/mL), median (IQR)	18.0 (7.8-41.8)	6	29.5 (9.5-86.3)	4	.43
Positive SPT result (no.)	0 of 15 (0%)	15	0 of 7 (0%)	13	1.00

Factors were investigated at the first hospital visit, whereas SPT was performed at the time of OFC.

*IQR*, Interquartile range.

∗ Statistically significant.

**Table III:** Diagnostic rate based on number of symptomatic episodes

No. of episodes	FPIES group (n = 30)	NA group (n = 20)	Diagnostic rate (%)
1	2	3	40
2	13	11	54
3	9	3	75
≥4	6	2	75
Missing data	0	1

**Table IV:** Specific IgE positivity rate

Specific IgE	FPIES group (n = 30)		NA group (n = 20)	
	Missing data (n)		Missing data (n)	
Hen’s egg yolk–specific IgE	2 of 5 (40%)	4	4 of 8 (50%)	4
Hen’s egg white				
Hen’s egg white–specific IgE	—	—	1 of 1 (100%)	0
Ovomucoid-specific IgE	—	—	0 of 1 (0%)	0
Soy-specific IgE	0 of 5 (0%)	0	0 of 1 (0%)	1
Wheat-specific IgE	0 of 4 (0%)	0	0 of 1 (0%)	0
Cow’s milk				
Cow’s milk–specific IgE	0 of 1 (0%)	2	0 of 2 (0%)	1
Casein-specific IgE	0 of 1 (0%)	2	0 of 2 (0%)	1
Quail’s egg yolk–specific IgE	—	2	—	—
Fish-specific IgE	0 of 1 (0%)	1	—	—
Rice-specific IgE	0 of 1 (0%)	0	0 of 1 (0%)	0
Buckwheat-specific IgE	0 of 1 (0%)	0	—	—
Kiwifruit-specific IgE	0 of 1 (0%)	0	—	—
Banana-specific IgE	1 of 1 (100%)	0	—	—
Shellfish-specific IgE	0 of 1 (0%)	0	—	—

## Discussion

This multicenter retrospective case-control study showed the difficulty of establishing a correct diagnosis of FPIES based solely on clinical history. This study found that the absence of a history of asymptomatic ingestion of culprit foods is a factor that reduced the likelihood of FPIES. The strength of this study is that patients with clinically suspected FPIES were diagnosed on the basis of OFC with a specific protocol.

There was no significant difference in the intensity and number of symptomatic episodes between the FPIES and NA groups in this study. Previous reports examining several patients with FPIES who had undergone OFC could not find any risk factors for a positive OFC result.^14^ The number of symptomatic episodes showed no significant difference between the 2 groups. The numbers of patients ultimately diagnosed with FPIES were as low as 15 of 29 (52%) for those with 1 or 2 symptomatic episodes and 15 of 20 (75%) for 3 or more episodes. Therefore, the international guidelines, which allow definite diagnosis based on the history of only 2 symptomatic episodes and other features, could lead to many false-positive results.

FPIES could also not be denied, even if there were multiple instances of a history of asymptomatic intake. This finding might reflect the pathogenic mechanisms in the development of FPIES. In this study, most patients in the FPIES group had a history of asymptomatic intake, which was significantly more common than in the NA group (*P* = .03). The precedence of an asymptomatic intake in FPIES would be a feature different from those of IgE-mediated food allergy, which often arises in patients without having ever ingested the culprit food.^15^ It is undeniable that there is a threshold dose for inducing FPIES symptoms. In this study, however, the patients in the FPIES group often reacted to the culprit food even though they had ingested a reduced dose for fear of allergy (data not shown), which does not support the threshold hypothesis. In previous reports, the culprit foods were often tolerated multiple times before FPIES onset in patients.^7^^,^^16^ A meta-analysis for the efficacy of early egg introduction to prevent IgE-mediated egg allergy also indicated a potentially harmful effect in that the prevalence of egg-related FPIES in the early-introduction group was significantly greater than the predicted prevalence.^17^ These facts might suggest the presence of priming mechanisms in the development of FPIES, such as an accumulation of damage to the gastrointestinal tract after an intrauterine acquisition of immunity or an oral sensitization of the culprit food.

No patient with FPIES in our cohort had multiple food triggers, although previous studies conducted in the United States or Australia have reported that up to one-third of patients with FPIES react to multiple food groups.^6^^,^^7^^,^^9^ A chart review of 462 patients with FPIES from the United States showed that 27% of patients had multiple food triggers.^6^ They also reported cow’s milk, soy, and grains as potential associations for FPIES with multiple trigger foods. This is in agreement with the culprit foods of FPIES as reported by a study conducted in Australia, with 32% of patients having multiple food FPIES.^7^ In contrast, a multicenter study conducted in Spain and Italy and analyzing comprehensive dietary history reported a low incidence of multiple food FPIES (ie, 6.1%).^18^ The causative foods differed significantly from those in the United States or Australia, with fish (57%), egg (19.5%), and cow’s milk (15.6%) comprising the majority. Furthermore, only 1 of the 28 children with FPIES to cow’s milk (3.6%) also had FPIES to other food, and those with FPIES to grains (7 of 179) had no concomitant food allergies. In this study, the number of tested patients was highest in the order of hen’s egg yolk, soybean, wheat, and cow’s milk. In all, 11 types of causative foods were found in 30 patients. Our cohort was relatively small, and the proportions of those with FPIES to grains, cow’s milk, and soy were low, unlike those in the studies conducted in the United States and Australia, which might have resulted in the absence of cases of multiple food FPIES.

Notably, hen’s egg yolk was a leading cause of FPIES in our study (in 9 of 30 patients [31%]). Many epidemiologic studies have reported geographic differences in the causative foods of FPIES,^1^^,^^5^^,^^8^ and it is hypothesized that environmental factors are essential for the onset (eg, many fish and shellfish FPIES cases in Italy^19^ and a few soy FPIES cases in Israel^2^). Recently, hen’s egg yolk was identified as the leading cause of FPIES in Japan, replacing cow’s milk.^20^ The clinical features of hen’s egg FPIES are similar to those with other trigger foods.^10^^,^^21^ A case series of 10 patients with hen’s egg yolk FPIES showed that all the patients consumed the antigen before the first symptomatic episode.^21^ In contrast, studies conducted in Sweden and Australia have reported that 45% to 51% of patients with FPIES reacted after their first ingestion, with grains and cow’s milk as the most common trigger foods.^7^^,^^22^ On the basis of these findings, it cannot be denied that the necessity of asymptomatic ingestion might be a characteristic feature of hen’s egg yolk FPIES. A history of asymptomatic ingestion was more prevalent in the FPIES group than in the NA group if focusing on patients with only hen’s egg yolk FPIES (9 of 9 patients [100%] vs 9 of 13 [69%]) or even after excluding them (14 of 15 [93%] vs 4 of 6 [67%]), with no significant difference (see Tables E1 and E2 in the Online Repository at www.jaci-global.org). In previous studies, OFC was not performed in all patients. It is undeniable that the guardians of some patients who vomited after the first ingestion might have opted for removal of the culprit foods owing to fear of allergies, which might have affected the physician’s diagnosis. Thus, there is a paucity of data for a definitive conclusion on this issue.

There was also no significant difference between the FPIES and NA groups with atopic stature and history of atopic diseases. FPIES is defined as a non–IgE-mediated food allergy. In contrast, “atypical FPIES” has long been known and defined on the basis of the international guidelines; it leads to food-specific IgE positivity but presents only delayed gastrointestinal symptoms for specific food without immediate-type symptoms of allergy. A population-based study revealed that the rate of comorbid IgE-mediated food allergy was higher in patients with FPIES than in those without FPIES.^1^ Of the patients tested in our study, 3 of 20 (15%) were positive for specific IgE, but none showed IgE-mediated symptoms of allergy on OFC. The sensitized patients in the NA group also showed no IgE-mediated symptoms on OFC. There were no clinical differences due to the sensitization. Our data suggest that IgE sensitization and atopic background do not have predictive value for the diagnosis of FPIES.

This study has some limitations. First, the study’s principal limitation is its retrospective data collection. The presence of missing data would have been a potential source of bias (eg, data regarding asymptomatic ingestion history were missing from 6 patients in the FPIES group; thus, the observation regarding this factor might be biased). Second, patients with FPIES with extremely early spontaneous remission might have been misclassified, as our criteria classified the patients with a negative OFC result within 120 days before the last symptomatic episode as belonging to the NA group. The result of the statistical analysis remained the same when the interval was set at 90 days. Sensitivity analysis of all OFC-negative cases exceeding 120 days included in the FPIES group also showed a tendency for asymptomatic ingestion history to be higher in the FPIES group without significance. Although there is a paucity of data on the precise prognosis of FPIES, remission within 120 days might be exceptional in the light of previous reports. Various studies have reported on the age of remission of FPIES, with the age ranging from 6 months to several years.^8^^,^^14^^,^^23^, ^24^, ^25^, ^26^ Third, 3 patients with a negative OFC result were classified as belonging to the FPIES group because they could not tolerate the culprit food after OFC. Barni et al also reported that 4.5% of patients with a negative OFC result at the hospital later (after they had returned home) experienced a reaction to the culprit food in amounts equal or higher than those administered at the hospital.^27^ Thus, we included these patients in the FPIES group in this study. One of these patients had a positive OFC result for remission confirmation after 6 months (data not shown). Excluding these 3 patients from the FPIES group did not change the statistical analysis results. Finally, our open OFC protocol might also be subject to patient bias. In this study, the patients’ psychogenic interference was unlikely, as most of the patients were infants or toddlers. The doctors’ interpretation bias was also minimized because our diagnostic criteria required protracted vomiting as the objective symptom.

In summary, our retrospective case-control study showed that there were no predictive background factors related to OFC positivity other than a history of asymptomatic ingestion. The absence of a history of asymptomatic ingestion was a negative predictor for the diagnosis of FPIES. Our data based on OFC results showed the difficulty of diagnosing FPIES solely on the basis of clinical history. Thus, the conventional diagnostic criteria based on the history of 2 symptomatic episodes and other features could yield several false-positive results. Therefore, OFC is essential for a definitive diagnosis of FPIES. Caution is required in the generalizability of our findings, as our cohort had a unique characteristic (ie, predominance of hen’s egg yolk as a cause of FPIES). The conflicting features of FPIES reported in previous studies might have been due to the heterogeneity of the cohorts, as there is no definitive diagnostic method for FPIES. Further investigation is expected to clarify the risk factors, prognostic factors, and diagnostic markers for FPIES. By prospectively accumulating more extensive data on patients suspected of having FPIES with OFC, more appropriate indications for OFC and the diagnostic criteria for FPIES could be established.

**Clinical implications**

The definite diagnosis of FPIES should be based on OFC, as there are no predictors for OFC positivity other than a history of asymptomatic ingestion.

## Supplementary data

Supplemental Matreials
