# Supplementary material for: Predictors of initial oral food challenge outcome in food protein–induced enterocolitis syndrome
Source: J Allergy Clin Immunol Glob. 2022 Jul 11;1(3):122–7. doi: 10.1016/j.jacig.2022.05.004 (PMC10509941; doi:10.1016/j.jacig.2022.05.004)
Supplement: Supplemental Matreials [file mmc1.docx]

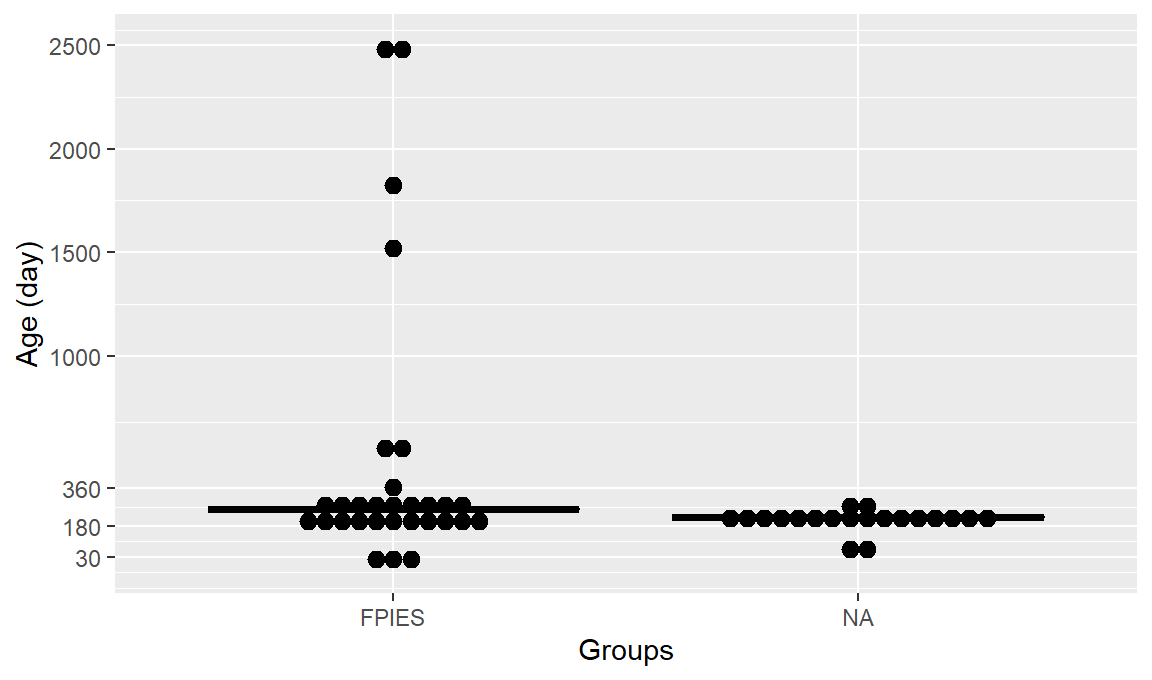


Figure S1. Age distribution of the two groups with median values (vertical lines). Each dot represents one study participant.

FPIES: food protein-induced enterocolitis syndrome, NA: no allergy


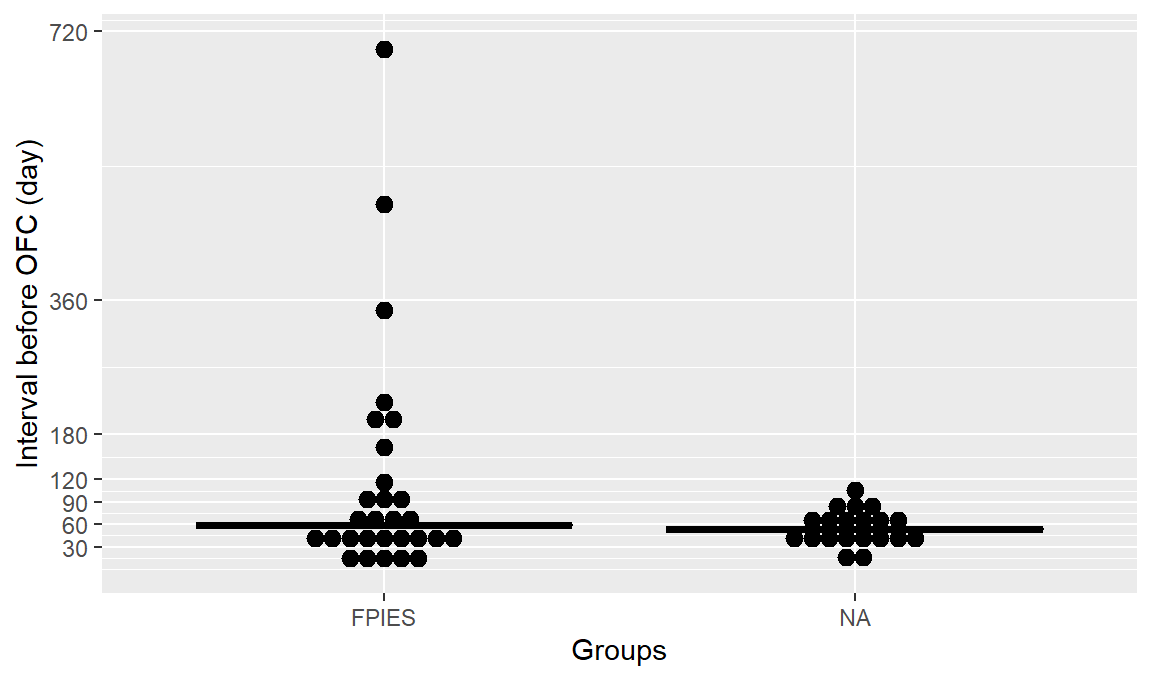


Figure S2. Distribution of the interval between the last symptomatic episode and the OFC with the median values shown as vertical lines. Each dot represents one study participant.

OFC: oral food challenge, FPIES: food protein-induced enterocolitis syndrome, NA: no allergy

Table S1. Clinical factors associated with FPIES^†^ (Only hen’s egg yolk FPIES cases included)

|  | FPIES group  (n = 9) | |  | No allergy group  (n = 13) | | |  |
| --- | --- | --- | --- | --- | --- | --- | --- |
|  |  | Missing data (n) |  |  | Missing data (n) |  | *P-*value |
| Age at the first episode (day), median (IQR) ^‡^ | 231 (216–268) | 0 |  | 221 (212–247) | 0 |  | 0.48 |
| Interval between the last symptomatic episode and the ^§^OFC (day), median (IQR) | 42 (31–85) | 0 |  | 57 (48–81) | 0 |  | 0.44 |
| Asymptomatic ingestion history (n) | 9 (100%) | 0 |  | 9 (69%) | 0 |  | 0.11 |
| Symptomatic episodes (n), median (IQR) | 2 (2–2) | 0 |  | 2 (2–2) | 0 |  | 0.42 |
| – Ingestion to onset interval (hours), median (IQR) | 2 (2–3) | 0 |  | 2 (1–2) | 0 |  | 0.33 |
| – Number of vomiting (n), median (IQR) | 3 (2–4) | 0 |  | 3 (1–3) | 0 |  | 0.84 |
| – Accompanied by diarrhea (n) | 2 (22%) | 0 |  | 2 (15%) | 0 |  | 1.00 |
| – Accompanied by bloody stool (n) | 0 (0%) | 0 |  | 0 (0%) | 2 |  | 1.00 |
| ^¶^IgE-mediated allergy to other than the culprit food (n) | 3 (33%) | 0 |  | 1 (7.7%) | 0 |  | 0.26 |
| Atopic dermatitis (n) | 6 (67%) | 0 |  | 5 (38%) | 0 |  | 0.39 |
| Family history |  |  |  |  |  |  |  |
| – Atopic dermatitis (n) | 0 (0%) | 0 |  | 4 (31%) | 0 |  | 0.11 |
| – Bronchial asthma (n) | 4 (44%) | 0 |  | 3 (23%) | 0 |  | 0.38 |
| Total IgE (IU/mL), median (IQR) | 12 (6.8–31) | 1 |  | 27 (11–67) | 3 |  | 0.29 |
| Positive ^††^SPT (n) | 0 (0%) | 4 |  | 0 (0%) | 7 |  | 1.00 |

Factors were investigated at the first hospital visit, whereas SPT was performed at the time of OFC.

^†^FPIES, food protein-induced enterocolitis syndrome; ^¶^IgE, immunoglobulin E; ^‡^IQR, interquartile range; ^§^OFC, oral food challenge; ^††^SPT, skin prick test

*Statistically significant

Table S2. Clinical factors associated with FPIES^†^ (with cases of hen’s egg yolk FPIES excluded)

|  | FPIES group  (n = 21) | |  | No allergy group  (n = 7) | | |  |
| --- | --- | --- | --- | --- | --- | --- | --- |
|  |  | Missing data (n) |  |  | Missing data (n) |  | *P-*value |
| Age at the first episode (day), median (IQR) ^‡^ | 264 (196–539) | 0 |  | 220 (196–226) | 0 |  | 0.16 |
| Interval between the last symptomatic episode and the ^§^OFC (day), median (IQR) | 62 (43–196) | 1 |  | 43 (36–62) | 0 |  | 0.17 |
| Asymptomatic ingestion history (n) | 14 (93%) | 6 |  | 4 (66%) | 1 |  | 0.18 |
| Symptomatic episodes (n), median (IQR) | 3 (2–4) | 1 |  | 2 (2–3) | 0 |  | 0.35 |
| – Ingestion to onset interval (hours), median (IQR) | 2 (2–3) | 4 |  | 2 (1–2) | 0 |  | 0.16 |
| – Number of vomiting (n), median (IQR) | 3 (1–5) | 1 |  | 3 (2–4.5) | 0 |  | 1.00 |
| – Accompanied by diarrhea (n) | 4 (20%) | 1 |  | 2 (29%) | 0 |  | 0.63 |
| – Accompanied by bloody stool (n) | 1 (5%) | 1 |  | 0 (0%) | 1 |  | 1.00 |
| ^¶^IgE-mediated allergy to other than the culprit food (n) | 2 (10%) | 1 |  | 0 (0%) | 0 |  | 1.00 |
| Atopic dermatitis (n) | 6 (32%) | 2 |  | 3 (43%) | 0 |  | 0.66 |
| Family history |  |  |  |  |  |  |  |
| – Atopic dermatitis (n) | 4 (21%) | 2 |  | 4 (57%) | 0 |  | 0.15 |
| – Bronchial asthma (n) | 5 (26%) | 2 |  | 2 (29%) | 0 |  | 1.00 |
| Total IgE (IU/mL), median (IQR) | 22 (8.8–63) | 5 |  | 35 (10–113) | 1 |  | 0.85 |
| Positive ^††^SPT (n) | 0 (0%) | 11 |  | 0 (0%) | 6 |  | 1.00 |

Factors were investigated at the first hospital visit, whereas SPT was performed at the time of OFC.

^†^FPIES, food protein-induced enterocolitis syndrome; ^¶^IgE, immunoglobulin E; ^‡^IQR, interquartile range; ^§^OFC, oral food challenge; ^††^SPT, skin prick test

*Statistically significant

Table S3. Clinical factors associated with FPIES^†^ (Sensitivity analysis)

|  | FPIES group  (n = 49) | |  | No allergy group  (n = 20) | | |  |
| --- | --- | --- | --- | --- | --- | --- | --- |
|  |  | Missing data (n) |  |  | Missing data (n) |  | *P-*value |
| Age at the first episode (day), median (IQR) ^‡^ | 262 (206–301) | 19 |  | 221 (211–243) | 0 |  | 0.11 |
| Interval between the last symptomatic episode and the ^§^OFC (day), median (IQR) | 156 (50–218) | 2 |  | 51 (42– 75) | 0 |  | 0.00 |
| Asymptomatic ingestion history (n) | 36 (90%) | 9 |  | 13 (68%) | 1 |  | 0.06 |
| Symptomatic episodes (n), median (IQR) | 2 (2–3) | 4 |  | 2.0 (2.0–2.5) | 0 |  | 0.10 |
| – Ingestion to onset interval (hours), median (IQR) | 2 (2–3) | 10 |  | 2 (1–2) | 0 |  | 0.15 |
| – Number of vomiting (n), median (IQR) | 3 (1–5) | 4 |  | 3.0 (1.8–4.3) | 0 |  | 0.92 |
| – Accompanied by diarrhea (n) | 9 (20%) | 4 |  | 4/20 (20%) | 0 |  | 1.00 |
| – Accompanied by bloody stool (n) | 2 (5%) | 9 |  | 0/17 (0%) | 3 |  | 1.00 |
| ^¶^IgE-mediated allergy to other than the culprit food (n) | 6 (13%) | 3 |  | 1/20 (5%) | 0 |  | 0.67 |
| Atopic dermatitis (n) | 19 (43%) | 5 |  | 8/20 (40%) | 0 |  | 1.00 |
| Family history |  |  |  |  |  |  |  |
| – Atopic dermatitis (n) | 5 (11%) | 4 |  | 8/20 (67%) | 0 |  | 0.02 |
| – Bronchial asthma (n) | 10 (22%) | 4 |  | 5/20 (25%) | 0 |  | 1.00 |
| Total IgE (IU/mL), median (IQR) | 18 (8–42) | 9 |  | 29.5 (9.5–86.3) | 4 |  | 0.43 |
| Positive ^††^SPT (n) | 1 (5%) | 30 |  | 0/7 (0%) | 13 |  | 1.00 |

Patients with negative OFC after 120 days or more from the first onset included into FPIES group. Factors were investigated at the first hospital visit, whereas SPT was performed at the time of OFC.

^†^FPIES, food protein-induced enterocolitis syndrome; ^¶^IgE, immunoglobulin E; ^‡^IQR, interquartile range; ^§^OFC, oral food challenge; ^††^SPT, skin prick test

*Statistically significant
